# Patterns and Predictors of Long-Term Glycemic Control in Patients with Type 2 Diabetes

**DOI:** 10.5402/2012/526824

**Published:** 2012-10-16

**Authors:** Mohsen Janghorbani, Masoud Amini

**Affiliations:** ^1^Department of Epidemiology and Biostatistics, School of Public Health, Isfahan University of Medical Sciences, Isfahan, Iran; ^2^Isfahan Endocrine and Metabolism Research Center, Isfahan University of Medical Sciences, Isfahan, Iran

## Abstract

*Aims/Introduction*. To describe patterns of long-term glycemic control among patients with type 2 diabetes in Isfahan, Iran and identify factors associated with glycemic control. *Methods*. During the mean (standard deviation (SD)) follow-up period of 8.4 (4.2) (range 1–18) years, 4,582 patients with type 2 diabetes have been examined to determine glycemic changes. Their glycated hemoglobin (GHb) at the last clinic visit was compared with the initial visit data. The mean (SD) age of participants was 49.3 (9.6) years with a mean (SD) duration of diabetes of 5.0 (5.1) years at initial registration. *Results*. Mean (SD) GHb was 8.7% (2.3) at baseline and 7.9% (1.9) at the study end and decreased by mean of 0.8% (95% confidence interval (CI) 0.74, 0.87; *P* < 0.001) and varied by the severity of baseline GHb. 74.6% at the initial visit versus 64.4% at the last clinic visit had GHb values above the target level of 7.0%. Using a stepwise multiple regression models, age, higher GHb, FPG, follow-up period, and number of follow-up visits increased and higher systolic BP and female gender significantly decreased the percent glycemic change. *Conclusions*. This study highlights that more than 64.4% of the patients have GHb values higher than 7.0% at last clinic visit andindicatesthe difficult challenges physicians face when treating their patients with type 2 diabetes. Clinical efforts should focus on more effective methods for glycemic control in diabetic patients.

## 1. Introduction

Type 2 diabetes is a progressive disease that requires ongoing increases in doses and complexity of hypoglycemic pharmacotherapy [1]. The goal of management of type 2 diabetes is improving the efficiency of diabetes care and to maintain blood glucose levels in the near-normal range. This is important to prevent sustained hyperglycemia with elevated glycated hemoglobin (GHb), which is associated with acute and long-term complications and to avoid recurrent episodes of hypoglycemia. Growing evidence that exists and improves glycemic control leads to a decrease in development and progression of vascular complications [2–4]. Despite the availability of evidence-based guidelines and vast knowledge about complications of diabetes, clinical goals for diabetes outcomes are not being achieved in routine care [2, 3, 5–9].

Although most studies have shown glycemic reduction to be associated with a significant improvement in diabetes control [2, 3, 5–12], few large-scale cohort studies evaluated long-term patterns and predictors of glycemic control and none in Iranian patients with type 2 diabetes receiving routine care. Information on predictors of glycemic change can lead to identification of patients who may have more difficulty controlling their diabetes. In light of the known benefits of glycemic reduction on type 2 diabetes management, greater insight into patterns of long-term glycemic change in diabetes, along with patient and treatment characteristics associated with glycemic change, is urgently needed. 

This study therefore used routinely collected data from a clinical information system for diabetes at Isfahan Endocrine and Metabolism Research Center, Iran, over a mean 8.4 year to describe patterns of long-term glycemic change in patients with type 2 diabetes receiving routine care and identifies factors associated with glycemic change. 

## 2. Patients and Methods

### 2.1. Study Area

 Our investigation was conducted in Isfahan, a very large area situated in central Iran, located on 1,590meter height above sea level, between latitudes 30 and 34 degrees north of the equator and longitude 49–55 degrees east, with a population of almost four and half million (4,559,256 in 2006 (men 2,335,399, women 2,223,857)) and a high proportion of young people. The total area is 107,029 Km^2^. The climate is dry temperate and quite wide temperature differences between the summer and the winter with a mean daily temperature of 3.0°C in January and February, 29.0°C in July and August, and 16.5°C in September and October. The population structure and socioeconomic status of Isfahan are similar to the rest of the country. Private physicians and hospitals, district health centers and government, and university hospitals and clinics provide the health services. 15 endocrinologists and 3 diabetes center serve the study area. Residency in remote and mountain areas and economical status may affect accessibility to the endocrinological expertise. 

### 2.2. Data Source

 The recruitment methods and examination procedures of the Isfahan Endocrine and Metabolism Research Center out patient clinics have been described before [13]. Briefly, clinical data are collected for all consecutive patients at the first attendance and at review consultations (usually annually) using standard encounter forms. These include an examination of ocular fundus, lens, limbs, blood pressure (BP), construction of a problem list by the clinician, measurement of height, weight, fasting plasma glucose (FPG), GHb, urine protein, triglyceride, cholesterol, and serum creatinine, and reporting of smoking as part of a completed questionnaire on demography, family history, and smoking by the patient. 

 All patients were referred for nutritional and weight management program after the start of the therapy by qualified nutritionists to evaluate the patient and if necessary recommend weight management program. A computerized patient registry provided data on patient characteristics, medications, and laboratory values. 

### 2.3. Patients

Between 1992 and 2010, a total of 14,243 patients with type 1 and type 2 diabetes were registered in the system. Women with diabetes diagnosed only during pregnancy were excluded. However, this study uses data only for 4,582 (1,725 (37.6%) men and 2,857 (62.4%) women) patients with type 2 diabetes who had at least one subsequent review since registration at baseline and for whom complete data were available. Attendees at the follow-up visit did not differ significantly from nonattendees regarding baseline GHb and high-density lipoprotein (HDL) cholesterol. However, nonattendees who were older (52.8 year versus 49.3 year, *P* < 0.001) had slightly higher systolic BP (128.4 mmHg versus 122.4 mmHg, *P* < 0.001), diastolic BP (79.0 mmHg versus 75.0 mmHg, *P* < 0.001), cholesterol (225.0 mg/dL versus 213.9 mg/dL, *P* < 0.001), triglyceride (233.2 mg/dL versus 218.7 mg/dL, *P* < 0.001), creatinine (1.0 *μ*M/L versus 0.9 *μ*M/L, *P* < 0.001), low-density lipoprotein (LDL) cholesterol (143.9 mg/dL versus 128.3 mg/dL, *P* < 0.001), FPG (206.7 mg/dL versus 184.6 mg/dL, *P* < 0.001), and duration of diabetes (6.8 year versus 5.0 year, *P* < 0.001) but lower levels of body mass index (BMI) (27.4 kg/m^2^ versus 28.2 kg/m^2^, *P* < 0.001) and weight (69.9 kg versus 71.7 kg, *P* < 0.001).

Predictors of GHb (measured by spectrophotometer) change were assessed using the following data from the patient's registration consultation: gender, age at diagnosis, age, educational level, duration of diabetes (the time between diagnosis and the baseline examination), BMI (weight/height^2^ (kg/m^2^)), smoking status (never, current), FPG, serum creatinine, triglyceride, cholesterol, HDL (measured using standardized procedures), LDL (calculated by the Friedwald equation [14] provided that total triglyceride did not exceed 400 mg/dL), and BP (systolic and diastolic) at registration. 

 Height and weight were measured with subjects in light clothes and without shoes using standard apparatus. Weight was measured to the nearest 0.1 kg on a calibrated beam scale. Height was measured to the nearest 0.5 cm with a measuring tape. Height was assessed at baseline only. A physician measured the systolic and diastolic BPs of seated participants after subjects had been seated for 10 minutes by using a mercury sphygmomanometer and standard techniques. All clinical measurements at baseline and follow-ups were made using the same standardized protocol.

The study protocol was approved by the Institutional Review Board of Isfahan University of Medical Sciences, Iran. 

### 2.4. Definitions

 GHb is recognized as the measure of glycemic control. Percent of GHb change was determined by taking the difference between the baseline and last measured GHb and divided that by patient's baseline GHb. A GHb level of <7% was used to indicate optimal glycemic control; this benchmark was established by the American Diabetes Association (ADA) as the recommended target [7]. GHb level of >9.5% was used to indicate poor glycemic control; this benchmark was established by the Healthcare Effectiveness Data and Information Set (HEDIS) [8]. A GHb level >9.5% is markedly hyperglycemic. Smoking was estimated from self-report and categorized in current and nonsmokers. Smoking status was assessed at baseline only. The physician defined the type of diabetes according to the ADA criteria [10]. 

### 2.5. Analysis

 Statistical methods used included the Student's *t*-test, Chi-square test, stepwise multiple linear regression model to test associations between baseline variables and percent GHb change. Forward stepwise multiple regression analysis was developed to determine independent predictors of percent GHb change using the SPSS, version 18.0 for Windows computer package (SPSS Inc., Chicago, IL, USA) which simultaneously adjusts for other covariates. For this analysis, age, FPG, systolic BP, total cholesterol, triglyceride, BMI, duration of diabetes, follow-up period, and number of follow-up visits were included as continuous variables. Percent GHb change was included as a continuous variable in its original form. The percentage of GHb change in either direction was used, with reduction of GHb having a positive value and increase in GHb having a negative value. The purpose was to determine the significance of the change over the continuum from maximum GHb reduction to maximum increase. Gender was entered as dichotomous variables. Therapeutic regimen (diet, oral agent, and insulin), and educational level (less than high school, high school, and college graduate) were included as trichotomous variable. In addition, multiple logistic regression involving same covariates was used to calculate the odds ratios of having poor GHb (>9.5%) control and to evaluate each measured variables independent association with poor glycemic control. Age-adjusted means were calculated and compared using general linear models. All statistical tests were two sided and *P* < 0.05 was considered statistically significant.

## 3. Results

### 3.1. Characteristics

 Patients had mean (SD) duration of diabetes 5.0 (5.1) years and mean age of 49.3 (9.6) years at baseline. The average time of follow-up was 8.4 (4.2) years (range 1–18 year). The average follow-up visits were 20.9 (18.0) times (range 2–114 visits). 22.7% of men and 1.6% of women were smoking at the baseline. The age-adjusted mean (SD) BMI was 26.8 (3.9) kg/m^2^ in men and 29.1 (4.5) in women. 

Population characteristics at baseline and last follow-up visit are presented in Table 1. Patients at the last clinic visit had higher weight, BMI, creatinine, diastolic BP and had lower FPG, GHb, triglyceride, cholesterol, and LDL than at baseline (*P* < 0.001). Frequency of insulin use was higher at last clinic visit, whereas frequency of hypoglycaemic medication and diet was lower at the last visit. Half (48.7%) of all patients were using hypoglycemic medication and 39.9% were treated with insulin (including 26.1% who used both insulin and oral agents) by the last visit. 

### 3.2. Changing GHb over Time

The mean (SD) GHb was 8.7% (2.3) at baseline and 7.9% (1.9) at the study end and decreased by mean of 0.8% (95% confidence interval (CI) 0.74, 0.87) over mean 8.4 years (*P* < 0.001). Of the 1,429 patients who had GHb >9.5% at initial registration, 921 (64.5%) subsequently reverted to GHb <9.5% (*P* < 0.001). Of the 1,987 patients who had GHb 7.0%–9.5% at initial registration, 590 (29.7%) improved to GHb <7.0% (*P* < 0.001).

On the other hand, of the 1,163 patients who had GHb <7.0% at baseline 418 (35.9%) subsequently worsen to GHb >7.0%. Of the 1,987 patients with GHb 7.0%–9.5% at initial registration, 263 (13.2%) subsequently progress to GHb >9.5%. This was lower than the rates of worsening seen for GHb <7.0%. 

Table 2 compares age-adjusted baseline characteristics of the 1,164 (25.4%) participants with ideal/optimal control (GHb <7.0%), 1,988 (43.4%) with suboptimal control (GHb 7.0%–9.5%) and 1,430 (31.2%) with poor control (GHb >9.5%). The three groups were significantly different with respect to baseline treatment, weight, height, systolic BP, follow-up, number of follow-up visits, and duration of diabetes, FPG, GHb, triglyceride, LDL, and cholesterol. Those with GHb >9.5% had a higher follow-up, duration of diabetes, FPG and GHb, but lesser number of follow-up visits. GHb at baseline was lower in those who subsequently had higher decreased GHb. 

### 3.3. Predictors of Changing GHb

**Figure 1:**
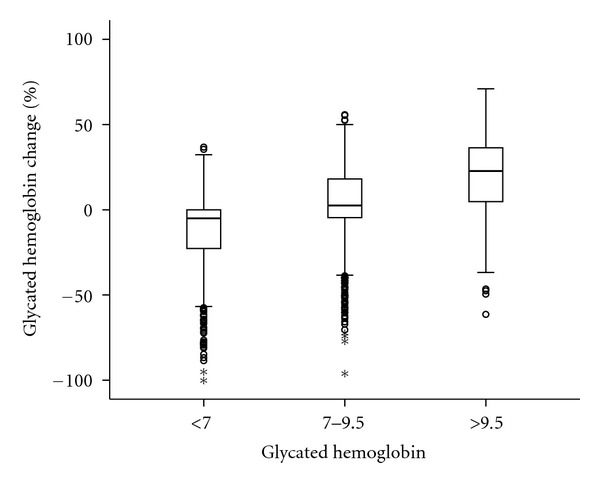
Glycated hemoglobin (GHb) change after 8.4-year follow-up by GHb category.

 The average 8.4-year GHb change for the entire population was 5.7 (95% CI: 5.0, 6.4) percentage points. The age-adjusted mean change in GHb varied by level of baseline GHb (Figure 1): a −11.5 (95% CI: −12.7, −10.4) percentage point increase in patients with GHb <7.0%, a 4.1 (95% CI: 3.2, 5.0) percentage point reduction for patients with GHb 7.0%–9.5%, and a 22.0 (95% CI: 20.9, 23.0) percentage point reduction for patients with GHb >9.5% (all changes significant at *P* < 0.001). With increasing GHb categories at baseline, patients were more likely to decreased GHb than those with lower GHb, indicating that patients with poorer GHb at baseline have a greater tendency to decrease it. 

Table 3 describes the age-adjusted associations of patient characteristics with percent GHb change. Characteristics associated with greater GHb reduction include male sex, higher education and longer duration of diabetes, insulin treatment regimen, smoker, higher FPG at the baseline and older at registration and diagnosis. When the patients were classified according to different therapeutic regimens, a difference in GHb change was observed. A higher proportion of those who used insulin or hypoglycemic medications decreased GHb. A small decrease in GHb occurred when patients were treated with diet. 

Percent of GHb change was slightly positively correlated with age (*r* = 0.082,  *P* < 0.001), age at diagnosis of diabetes (*r* = 0.048,  *P* < 0.01), duration of diabetes (*r* = 0.06,  *P* < 0.001), FPG (*r* = 0.177,  *P* < 0.001), GHb (*r* = 0.563,  *P* < 0.001), follow-up duration (*r* = 0.139,  *P* < 0.001), number of follow-up visits (*r* = 0.147,  *P* < 0.001), cholesterol (*r* = 0.047,  *P* < 0.01), LDL (*r* = 0.072,  *P* < 0.05) and negatively correlated with BMI (*r* = −0.066,  *P* < 0.001) and HDL (*r* = −0.05,  *P* < 0.05) at baseline.

The percent of GHb change was also analyzed with multivariate regression analysis. Age (*β* = 0.081, *P* < 0.001) and higher GHb (*β* = 6.281, *P* < 0.001), FPG (*β* = 0.174, *P* < 0.001), follow-up (*β* = 0.057, *P* < 0.01) and number of follow-up visits (*β* = 0.109, *P* < 0.001) increased and higher systolic BP (*β* = −0.044, *P* < 0.01) and female (*β* = −0.042, *P* < 0.01) significantly decreased the percent of GHb change. The overall contribution (*R* ^2^) of these factors was less than 7.0%, therefore the predictors for percent of GHb change during mean 8.4-year follow-up are yet unresolved.

The strength and statistical significance of the relationship of baseline characteristics to GHb >9.5% were also tested by multiple logistic regression. Findings of this analysis show that younger age (OR 0.99, 95% CI: 0.98, 0.99), higher FPG (OR 1.002, 95% CI: 1.001, 1.004), GHb (OR 1.35, 95% CI: 1.30, 1.41) at baseline, and higher follow-up duration (OR 1.10, 95% CI: 1.07, 1.13) significantly increased and treatment with oral agent (OR 0.57, 95% CI: 0.37, 0.88) and with insulin (OR 0.48, 95% CI: 0.29, 0.79), higher education (OR 0.48, 95% CI: 0.33, 0.70), and number of follow-up visits (OR 0.97, 95% CI: 0.96, 0.97) significantly decreased the risk of having GHb values >9.5% compared with GHb ≤9.5% (Table 4). 

## 4. Discussion 

In this large cohort study of patients with type 2 diabetes who received routine care we observed that 35.6% of patients with type 2 diabetes achieved the clinical goals for diabetes during average 8.4 years. These findings suggest that a considerable proportion of patients with type 2 diabetes in Isfahan, Iran, are not well controlled. The estimate of poor GHb control, defined as GHb >9.5%, in our study cohort was 31.2% at baseline and 18.0% at the study end. This is much lower than 32.7% reported by McBean et al. among American elderly managed care beneficiaries [15], 33.4% reported by National Committee for Quality Assurance for Medicare managed care beneficiaries of all ages and 42.5% reported for the commercial care plans [16]. Using the Third National Health and Nutrition Examination Survey, which includes patients with type 2 diabetes treated in both managed care and fee-for-service settings, Harris et al. [17] reported that poor glycemic control, defined as GHb >8%, was 37.1%. In a study of family practice patients with type 2 diabetes in Canada 74.3% of patients were not at target [18]. We also found that in the cohort of patients with type 2 diabetes, there was a decrease in GHb at average 8.4-year follow-up, with the most significant GHb reduction observed in higher GHb categories at baseline. In addition, we found that long-term GHb change was associated with a number of factors, including age and higher GHb, FPG, follow-up duration, and number of follow-up visits significantly increased and higher systolic BP and female gender significantly decreased the percent glycemic change. These factors remained significant even after adjusting for a wide range of patient characteristics. Younger age, treatment with oral agent and insulin, lower education and higher FPG, GHb, follow-up duration, and number of follow-up visits were disproportionately represented among those in poor glycemic control. These data can serve an important role in alerting physicians about the minimal amount of GHb reduction accomplished in most patients with type 2 diabetes and in designing interventions to support GHb reduction after diagnosis of diabetes. 

Although interventional studies have shown that GHb reduction produces improvement in microvascular complications due to this disease, few cohort studies describe the clinical course of GHb changes after diagnosis of type 2 diabetes in patients receiving routine care [1–5], and the results are inconsistent. Best et al. reported over the course of 5 years, GHb increased by an average of 0.22% in patients with type 2 diabetes [19]. Ott et al. in diabetes in Germany study reported that mean GHb was 6.98% at base line and 7.03% at the study end and increased by an average of 0.05% over 3.7 years [20]. However, their study populations are very different with present study in regard to age, gender, race, methodology, access to medical care, and genetic background. Therefore, it is difficult for us to compare our results with these studies. Nevertheless, our data, as well as the data obtained in Best et al. and Ott et al. and others [2, 3, 5–9] indicate that long-term GHb change was, on average, minimal. However, the pattern of GHb change in type 2 diabetes is not well recognized and may be quite variable according to patients' characteristics. While the mean GHb at last clinic visit in our study was higher than ADA goals and a large percentage of patients have not achieved the targeted values, type 2 diabetes in Isfahan, Iran received an acceptable level of treatment for hyperglycemia. However, there remains room for improvement. Data from the United Kingdom Prospective Diabetes Study suggested that a 1% reduction in mean GHb results in 21% fewer deaths, 14% fewer myocardial infections, and a 37% decrease in microvascular complications at the population level [21]. We reported a 0.8% reduction in mean GHb, which might translate to 17% fewer deaths, 11% fewer myocardial infarction, and 30% fewer microvascular complications at the population level. It is plausible that further population-level improvements in these outcomes could be achieved through better glycemic managements. 

We found that BMI was not predictive of poor glycemic control. Obesity was not related to poor glycemic control, probably because patients with type 2 diabetes, including patients in good glycemic control who have gained weight and patients with poor glycemic control who have lost weight due to disease process. A study among public-hospital patients with type 2 diabetes demonstrated no relationship between BMI and degree of glycemic control [22].

The reasons for relationship between age, gender, and GHb level are unclear. Younger age was associated with poorer glycemic control because their duration of diabetes was greater than those diagnosed at older ages. Longer duration of diabetes is known to be associated with poor glycemic control [17], possibly due to progressive impairment of insulin secretion because of beta cell failure [23], compounding the adverse effects of insulin resistance. Type 2 diabetes often has an insidious onset, making it difficult for studies to assess how GHb changes with respect to duration of diabetes. It is possible also that patients with higher GHb levels died younger because of diabetes related vascular complications, and/or that older patients are more adherent to recommendations for meal planning and more complaint with pharmacological regimen compared with younger patients. Glasgow et al. [24] reported that older patients with diabetes had significantly better scores than younger patients on an instrument that measured barriers to testing of glucose levels, regular physical activity, healthy low-fat eating, and compliance with medications. Additional studies have reported that older patients tend to keep their follow-up appointments more regularly than younger patients, and that patients who keep their follow-up appointments tend to achieve better glycemic control [25]. The reason(s) for this gender difference in GHb change has not been explored.

Our analysis also showed that treatment with oral agent and insulin at baseline was associated with a better glycemic control. This is expected, because patients with more sever hyperglycemia are more likely to have been prescribed oral agent and/or insulin compared with patients with milder hyperglycemia. 

We found that long-term GHb change was an increase with number of follow-up visits. These patients are more likely to consult a physician on a regular basis and, therefore, are more likely to be offered appropriate treatment. 

Our findings are consistent with previously published findings that education was associated with better glycemic control [26]. There are several potential reasons why improvements in glycemic control may have been concentrated among more educated populations. More educated people may have better access than lesser educated individuals to the type of integrated, comprehensive medical care that individuals with diabetes need in order to successfully manage their illness. Patients with diabetes who are more educated may have been better able to obtain and understand new information related to diabetes treatment compared with patients with diabetes who are less educated. There also is evidence that people who are more educated adopt medical technologies more rapidly than people who are less educated [27].

The strengths of this study include the large size, long-term follow-up, sample consisting of both men and women of a wide age range, and detailed information on potential confounding factors. Selection and information bias were unlikely because of the prospective design and high rate of follow-up. These real-life data reflect actual treatment pattern and allow for observation of patients over time. Several limitations of this study should be considered when interpreting our results. We used patient GHb only at the baseline and at last follow-up visits. We could not rule out the possibility of residual confounding because of unmeasured or inaccurately measured covariates. Our study was limited by possible selection bias by restricting the study to patients alive during the whole study period. The possibility exists that the people with diabetes who had the most severe disease or who were in the least good control died before the end of the study and were not included in the sample. This may result in overly optimistic estimates of glycemic control. Loses to follow-up are the major source of bias in longitudinal studies. The slight difference between attendees and nonattendees with regard to age, BP, lipid profile, FPG, duration of diabetes, and BMI might restrict generalizability of our findings. This is the first report of diabetes outcomes measures in routine care in a developing country and provides new data from Iran which has been underrepresented in past studies. 

In conclusion, this study highlights the difficult challenges physicians face when treating their patients with type 2 diabetes, such as the low frequency of achieving a clinically significant amount of GHb reduction. Although this population of Iranian type 2 diabetes had small glycemic change over mean 8.4 years and more than 64.4% of the patients have GHb values higher than 7.0%, type 2 diabetes in Isfahan, Iran received an acceptable level of treatment for hyperglycemia, though not optimal. 

## Figures and Tables

**Table 1:** Characteristics of 4,852 patients with type 2 diabetes mellitus at baseline and last follow-up visit.

Characteristics	Mean (SE)		Difference (95% CI)
	Baseline	Last follow-up
Age (yr)	49.3 (0.17)	56.2 (0.19)	−7.1 (−7.25, −6.91)*
Age at diagnosis (yr)	44.3 (0.14)	—	—
Duration of diabetes (yr)	5.0 (0.08)	—	—
Follow-up duration (yr)	—	8.4 (0.06)	—
Number of follow-up visit	—	20.9 (0.27)	—
GHb change (%)	—	5.7 (0.36)	—
Weight (kg)	71.7 (0.18)	73.2 (0.18)	−1.5 (−1.69, −1.28)*
Height (cm)	159.4 (0.14)	—	—
BMI (kg/m^2^)	28.2 (0.07)	28.8 (0.07)	−0.6 (−0.69, −0.52)*
Systolic BP (mmHg)	122.4 (0.26)	123.0 (0.28)	−0.6 (−1.11, 0.06)
Diastolic BP (mmHg)	75.0 (0.18)	77.4 (0.16)	−2.4 (−2.85, −2.01)*
Fasting plasma glucose (mg/dL)	184.6 (1.02)	155.7 (0.87)	28.9 (26.57, 31.23)*
GHb (%)	8.7 (0.03)	7.9 (0.03)	0.8 (0.74, 0.87)*
Creatinine (*μ*M/L)	0.90 (0.009)	1.03 (0.007)	−0.13 (−0.15, −0.11)*
Triglyceride (mg/dL)	218.7 (2.19)	173.1 (1.53)	45.6 (41.58, 49.50)*
Cholesterol (mg/dL)	213.9 (0.71)	185.3 (0.60)	28.6 (27.17, 30.13)*
High-density lipoprotein (mg/dL)	44.9 (0.22)	44.8 (0.23)	0.1 (−0.40, 0.54)
Low-density lipoprotein (mg/dL)	128.3 (0.84)	104.4 (0.64)	23.9 (22.08, 25.70)*
Gender %			
Men	37.6	—	—
Women	62.4	—	—
Therapeutic regimen %			
Diet	20.9	11.4	9.5 (8.06, 11.10)*
Oral agent	67.7	48.7	19.0 (17.1, 21.00)*
Insulin	8.3	13.8	−5.5 (−6.80, −4.24)*
Insulin and oral agent	3.1	26.1	−23.0 (−24.50, −21.70)*
Education %			
Less than high school	58.7	—	—
High school	30.0	—	—
College graduate	11.3	—	—
Smoking %			
Nonsmoker	90.4	—	—
Current smoker	9.6	—	—
Glycated hemoglobin %			
<7%	25.4	35.6	−10.2 (−12.00, −8.28)*
7%–9.5%	43.4	46.4	−3.0 (−5.07, −0.10)*
>9.5%	31.2	18.0	13.2 (11.40, 14.90)*
Weight category %			
Normal weight (BMI < 25.0 kg/m^2^)	23.4	19.9	3.5 (1.79, 5.22)*
Overweight (BMI 25–29.9 kg/m^2^)	45.7	44.1	1.6 (−0.42, 3.72)
Obese (BMI ≥ 30.0 kg/m^2^)	30.9	36.0	−5.1 (−7.12, −3.19)*

*P < 0.00, CI: confidence interval.

**Table 2:** Age-adjusted comparison of baseline variables by glycemic control group in 4,852 patients with type 2 diabetes mellitus.

Characteristics	Mean (SE)		
	GHb < 7.0%	GHb 7.0%–9.5%	GHb ≥ 9.5%
Number (%)	1164 (25.4)	1988 (43.4)	1430 (31.2)
Age (yr)	49.5 (0.24)	49.3 (0.21)	48.7 (0.33)
follow-up (yr)	7.6 (0.10)	8.8 (0.09)	9.1 (0.15)***
Number of follow-up visit	19.9 (0.44)	22.7 (0.38)	18.3 (0.62)***
Duration of diabetes (year)	4.2 (0.12)	5.3 (0.10)	5.7 (0.17)***
Weight (kg)	72.3 (0.30)	71.4 (0.27)	71.0 (0.43)*
Height (cm)	159.9 (0.23)	159.1 (0.20)	159.2 (0.32)*
BMI (kg/m^2^)	28.3 (0.11)	28.2 (0.10)	28.1 (0.16)
Systolic BP (mmHg)	121.6 (0.41)	122.7 (0.36)	123.2 (0.57)*
Diastolic BP (mmHg)	74.5 (0.30)	75.2 (0.26)	75.4 (0.43)
Fasting blood glucose (mg/dL)	167.1 (1.66)	187.1 (1.46)	213.1 (2.34)***
GHb (%)	7.7 (0.05)	8.9 (0.05)	10.2 (0.07)***
Creatinine (*μ*M/L)	0.91 (0.02)	0.90 (0.01)	0.90 (0.02)
Triglyceride (mg/dL)	209.4 (3.67)	219.7 (3.20)	234.8 (5.15)***
Cholesterol (mg/dL)	209.6 (1.19)	214.8 (1.04)	220.1 (1.67)***
GHb change (%)	−11.6 (0.60)	4.1 (0.46)	22.0 (0.54)***
HDL (mg/dL)	44.7 (0.37)	45.0 (0.32)	45.2 (0.60)
LDL (mg/dL)	125.0 (1.37)	129.8 (1.20)	132.0 (2.28)**
Gender %			
Men	39.9	36.3	36.8
Women	60.1	63.7	63.2
Therapeutic regimen %			
Diet	28.2	17.7	15.0***
Oral agent	64.6	69.8	68.4***
Insulin	7.2	12.4	16.6***
Education %			
Less than high school	51.7	61.8	64.9***
High school	32.3	28.2	29.9***
College graduate	16.0	10.0	5.2***
Smokers %	9.1	9.9	9.9
Weight category %			
Normal weight (BMI < 25 kg/m^2^)	21.8	23.0	26.9*
Overweight (BMI 25–29.9 kg/m^2^)	48.1	45.7	41.1*
Obese (BMI > 30 kg/m^2^)	30.1	31.3	32.0*

Age-adjusted means were calculated using general linear models. Comparison across all three groups. *P < 0.05, **P < 0.01, ***P < 0.001, CI: confidence interval; GHb: glycated hemoglobin.

**Table 3:** Age-adjusted associations of patient characteristics at baseline with percent of glycated hemoglobin (GHb) change at average 8.4 year, Isfahan, Iran.

Variables	Age-adjusted mean (SE) percent GHb change			
	Entire group	GHb < 7.0%	GHb 7.0%–9.5%	GHb > 9.5%
Number (%)	4582 (100.0)	1164 (25.4)	1988 (43.4)	1430 (31.2)
Gender				
Men	7.1 (1.59)	−11.2 (1.01)	5.7 (0.77)	22.7 (0.87)*
Women	4.9 (0.45)	−11.7 (0.74)	3.2 (0.57)	21.6 (0.69)*
Age at registration (yr)				
<40	3.8 (0.92)	−11.5 (1.52)	3.5 (1.23)	19.6 (1.52)*
40–49	4.1 (0.58)	−11.7 (0.95)	2.5 (0.76)	21.1 (0.91)*
50–59	6.7 (0.60)	−11.2 (1.04)	4.9 (0.77)	22.7 (0.89)*
60–69	9.2 (1.10)	−13.8 (2.14)	6.7 (1.39)	24.5 (1.55)*
≥70	10.3 (2.02)	−7.2 (3.51)	7.4 (2.51)	25.2 (2.82)*
Age at diagnosis (yr)				
<30	4.5 (1.59)	−13.8 (2.90)	1.9 (2.14)	19.3 (2.31)*
30–59	5.5 (0.38)	−11.3 (0.63)	3.7 (0.49)	22.0 (0.58)*
≥60	9.1 (1.54)	−15.3 (2.69)	11.5 (1.92)	23.9 (2.33)*
Duration of diabetes (yr)				
<5	4.6 (0.47)	−11.4 (0.72)	4.9 (0.60)	22.6 (0.78)*
5–7	6.3 (0.83)	−11.7 (1.50)	3.0 (1.04)	21.5 (1.18)*
8–11	7.4 (1.07)	−14.6 (2.15)	2.8 (1.35)	22.5 (1.44)*
≥12	9.3 (1.03)	−10.1 (2.57)	2.9 (1.40)	20.3 (1.33)*
Fasting blood glucose (mg/dL)				
<100	0.9 (1.70)	−9.2 (2.02)	2.5 (2.59)	26.5 (3.45)*
100–125	−0.2 (1.00)	−12.2 (1.25)	5.5 (1.37)	26.7 (2.41)*
≥126	7.0 (0.39)	−11.6 (0.74)	3.9 (0.50)	21.5 (0.56)*
Systolic BP (mmHg)				
<140	5.6 (0.40)	−10.7 (0.65)	4.0 (0.52)	21.6 (0.61)*
140–159	7.2 (0.97)	−15.1 (1.82)	5.3 (1.19)	23.5 (1.42)*
≥160	5.7 (1.57)	−19.4 (2.85)	2.4 (1.86)	23.4 (2.09)*
Diastolic BP (mmHg)				
<70	4.6 (0.67)	−9.9 (1.05)	5.5 (0.93)	18.6 (1.07)*
70–90	6.3 (0.48)	−10.9 (0.83)	3.9 (0.60)	22.8 (0.72)*
≥90	5.4 (0.89)	−16.7 (1.51)	3.3 (1.10)	23.7 (1.26)*
Therapeutic regimen				
Diet alone	2.8 (0.79)	−10.0 (1.14)	6.8 (1.04)	20.9 (1.72)*
Oral agent	6.5 (0.43)	−11.6 (0.75)	3.3 (0.54)	22.9 (0.62)*
Insulin	6.8 (1.07)	−17.5 (2.23)	3.5 (1.46)	18.9 (1.37)*
Education				
Less than high school	7.1 (0.48)	−13.9 (0.89)	3.9 (0.59)	22.6 (0.66)*
High school	2.7 (0.66)	−11.4 (1.04)	3.6 (0.90)	18.8 (1.16)*
College graduate	6.3 (0.99)	−6.2 (1.33)	7.4 (1.27)	28.0 (1.83)*
Smoking				
Nonsmoker	7.3 (0.48)	−13.7 (0.91)	4.2 (0.60)	22.7 (0.68)*
Current-smoker	8.3 (1.47)	−10.4 (3.08)	3.5 (2.01)	21.3 (2.04)*

Age-adjusted means were calculated using general linear models. Category definitions are based on ADA and HEDIS cut-offs [7, 8]. *P < 0.001.

**Table 4:** Findings of logistic regression analysis to determine predictors of poor glycemic control in 4,852 patients with type 2 diabetes mellitus.

Characteristics	OR (95% CI)
Age (yr)	0.99 (0.98, 0.99)*
BMI (kg/m^2^)	1.02 (0.99, 1.04)
Systolic BP (mmHg)	1.00 (0.99, 1.04)
Fasting plasma glucose (mg/dL)	1.002 (1.001, 1.004)***
GHb (%)	1.35 (1.30, 1.41)***
Duration of diabetes (yr)	1.01 (0.99, 1.03)
follow-up (yr)	1.10 (1.07, 1.13)***
Number of follow-up visit	0.97 (0.96, 0.97)**
Creatinine (*μ*M/L)	0.96(0.83, 1.12)
Triglyceride (mg/dL)	1.00 (1.00, 1.001)
Cholesterol (mg/dL)	1.00 (0.99, 1.002)
Gender	
Men	1.00
Women	1.02 (0.85, 1.24)
Therapeutic regimen	
Diet	1.00
Oral agent	0.57 (0.37, 0.88)*
Insulin	0.48 (0.29, 0.79)**
Education	
Less than high school	1.00
High school	1.12 (0.91, 1.37)
College graduate	0.48 (0.33, 0.70)***

Poor glycemic control is defined as a GHb level of >9.5% based on Health Care Effectiveness Data and Information Set (HEDIS) [8]. CI: confidence interval; OR: odds ratio. *P < 0.05, **P < 0.01, ***P < 0.001.
